# Facilitating clinical application of a dimensional model for personality disorders across the entire adult lifespan: thresholds for dysfunction and a multi-method approach

**DOI:** 10.3389/fpsyg.2026.1734213

**Published:** 2026-04-24

**Authors:** Morag Facon, Eva Dierckx, Sebastiaan P. J. van Alphen, Selinay Surer, Gina Rossi

**Affiliations:** 1 Personality and Psychopathology Research Group (PEPS), Department of Psychology, Vrije Universiteit Brussel (VUB), Brussels, Belgium; 2 Psychiatric Clinic, Alexianen Zorggroep Tienen, Tienen, Belgium; 3 Clinical Centre of Excellence for Personality Disorders in Older Adults, Mondriaan Mental Health Center, Heerlen/Maastricht, Netherlands; 4 Department of Clinical Psychological Science, Faculty of Psychology and Neuroscience, Maastricht University, Maastricht, Netherlands

**Keywords:** adult lifespan, dimensional classification, gender, multi-method, normative data, older adults, personality disorders

## Abstract

**Introduction:**

The classification of personality disorders (PDs) may be enhanced by dimensional models such as the Alternative Model for Personality Disorders (AMPD) and the ICD-11. However, their implementation in clinical practice remains limited, particularly among older adults, who are underrepresented in PD research. Given that personality pathology may vary across the lifespan, incorporating an age-sensitive perspective is essential when applying dimensional models.

**Methods:**

This study included a non-clinical sample of 754 participants aged 18 to 97 and a clinical sample of 102 older adult patients aged 65 and above. The discriminative validity of the LPFS-BF 2.0 and PID-5-BF + M was first assessed. Subsequently, age- and gender-specific normative thresholds were developed based on the non-clinical sample and evaluated within the clinical sample. Finally, self-report measures were compared with informant reports and clinical interview data (STiP-5.1) in the clinical older adult group.

**Results:**

Both the LPFS-BF 2.0 and PID-5-BF + M effectively differentiated between clinical and non-clinical participants. Age and gender influenced mean scores, supporting the need for adjusted normative thresholds. These thresholds successfully identified clinically elevated cases, particularly for the LPFS-BF 2.0 total score and the PID-5-BF + M domains of Negative Affectivity, Detachment, and Psychoticism. Moderate agreement was observed between self-reports, informant reports, and clinical interviews, although not all scales showed consistent correlations.

**Discussion:**

These findings highlight the importance of accounting for age and gender in the clinical application of dimensional PD models and support the use of multi-method assessment approaches.

## Introduction

The Diagnostic and Statistical Manual of Mental Disorders, 5th Edition and Text Revision (DSM-5 and DSM-5-TR) (American Psychiatric Association [APA], 2013, 2022) defines personality disorders (PDs) as 10 different categories, each defined by a set of criteria. Prior research and clinicians have however criticized these categories heavily, as they show problems with heterogeneity, reliability, co-morbidity and arbitrary thresholds (Mulder and Tyrer, 2018). Additionally, as the model’s criteria were primarily developed based on characteristics observed in adults aged 18–40, they may not adequately capture the manifestations of PDs in later life (van Alphen et al., 2015). For example, shifts in social roles, occupational involvement, and family responsibilities can significantly alter the expression of PD traits with age, which is not represented in the current criteria (van Alphen et al., 2015). Because of this, older adults currently experience both under- and overdiagnosis of PDs, leading to lack of appropriate treatment (van Alphen et al., 2015). Given that the global population is currently greying, this will form an increasingly large burden on mental health care systems (van Alphen et al., 2015).

Fortunately, emerging insights in personality research have paved the way for new methods of classifying PDs. More specifically, a paradigm shift has occurred from traditional, categorical systems to dimensional models has resulted in alternative approaches that provide more flexible and nuanced frameworks. Currently, two dimensional classification models for PDs exist: the Alternative Model for Personality Disorders (AMPD) in Section III of the DSM-5-TR (American Psychiatric Association, 2022) and the International Classification of Diseases, 11th Edition (ICD-11) (WHO, 2019). Both models conceptualize PDs as conditions characterized by impairments in personality functioning. The severity of these impairments is assessed on a dimensional scale, reflecting the overall level of dysfunction. Beyond this initial assessment, both AMPD and ICD-11 use maladaptive personality traits to describe the specific presentation of a disorder. In AMPD, this trait-based classification is a fundamental part of a two-step diagnostic process, whereas in ICD-11, traits serve as an additional (but not mandatory) descriptor. AMPD categorizes maladaptive traits into five broad domains, each further broken down into distinct facets. ICD-11, on the other hand, defines only five trait domains without further subdivision. Despite differences in structure, the models share four core maladaptive personality domains: Negative Affectivity, Detachment, Antagonism, and Disinhibition. However, they diverge in their fifth domain: AMPD includes Psychoticism, while ICD-11 instead incorporates Anankastia (American Psychiatric Association, 2022; WHO, 2019). By conceptualizing personality pathology along a continuum rather than through rigid diagnostic categories, these dimensional models offer a more flexible approach to understanding PDs across the lifespan (van Alphen et al., 2023). This is corroborated by psychometrical research that generally supports the use of these models both in younger and in older adult populations (e.g., Facon et al., 2025; Stone et al., 2021; Zimmermann et al., 2019).

The ongoing evolution of these dimensional models has yielded diverse operationalizations, primarily in the form of self-report instruments. Two of those instruments are the Level of Personality Functioning Brief Form 2.0 (LPFS-BF 2.0; Weekers et al., 2019) and the Personality Inventory for DSM-5 Brief Form Modified + (PID-5-BF + M; Bach et al., 2020). The LPFS-BF 2.0 evaluates personality functioning as conceptualized by the AMPD and aligns with the ICD-11 framework (Prevolnik Rupel et al., 2021), while the 36-item PID-5-BF + M captures combined trait domains (Negative Affectivity, Detachment, Antagonism, Disinhibition, Anankastia, and Psychoticism). These instruments have generated strong support across populations, including younger and older adults, making them appear suitable for use across the adult lifespan (Bach et al., 2020; Bach and Hutsebaut, 2018; Stone et al., 2021; Facon et al., 2023; Weekers et al., 2019). Due to their brevity, they may be applied for screening purposes, as a first step to identifying individuals with an indication of personality pathology who could benefit from a more extensive assessment. However, to ensure their utility and applicability in clinical practice with both younger and older adults, two critical elements remain to be tackled: age- and gender sensitive score interpretation and integration into a broader multi-method diagnostic framework.

First, in order to identify individuals in need of more assessment, specific cut-off points to interpret scores accurately must be provided. Regarding the PID-5-BF + M, no normative data has been developed yet. The instrument has however shown promising results with regards to discriminative validity, as an earlier study demonstrated its ability to differentiate levels of maladaptive traits across clinical versus non-clinical samples (Pires et al., 2023). For the LPFS-BF 2.0, normative data for adults aged 18–81 has been established in Denmark by Weekers et al. (2022), while Polish norms were developed by Juras et al. (2025). According to the Danish data, LPFS-BF 2.0 total scores of 26 and 31 correspond to 1.0 and 1.5 standard deviations above the latent mean, which are typically interpreted as reflecting mild (subclinical) and moderate (clinical) personality dysfunction, respectively. Polish norms are more conservative, with scores of 32 and 36 marking the respective norms. Despite some normative data already existing, it seems necessary to extend this research by developing age-sensitive thresholds. More specifically, previous research has shown that PDs can fluctuate throughout the entire lifespan and that, typically, older adults yield lower scores on both functioning and traits than younger adults (e.g., Facon et al., 2025; Videler et al., 2019). This suggests that functioning and traits may not be directly comparable between age groups, and that age sensitive thresholds are necessary for interpreting scores that capture these concepts, correctly. Besides age, it may also be important to account for gender and/or sex differences when establishing accurate normative data. Previous research has identified mean-level gender/sex differences in both personality functioning and maladaptive traits across both younger and older adult populations (e.g., Cavelti et al., 2025; Facon et al., 2025; Gomez et al., 2022; Le Corff et al., 2023), suggesting that normative thresholds also need to be adjusted for gender/sex.

Next, in addition to accurate normative data, these brief screenings must be embedded within a multi-method assessment framework. Once an initial screening indicates potential pathology, the results should be integrated with other sources, such as informant reports and semi-structured clinical interviews, to arrive at a comprehensive diagnostic formulation (Penders et al., 2020; Widiger, 2008). For an evidence-based assessment of a PD, it is recommended to first administer a self-report tool to screen for potential pathological personality aspects, followed by a semi-structured interview that further details and verifies the PD (Widiger et al., 2024). Including informant reports can also improve the validity of the assessment (Widiger et al., 2024). Such a stepwise, multi-source approach benefits clinical practice by enhancing efficiency and also provides a more comprehensive understanding of the PD, as each method has been shown to provide unique information (Widiger et al., 2024).

Self-reports are cost- and time-efficient and align with much of the existing research on personality assessment (Huprich et al., 2011). They are particularly sensitive to internal traits and experiences that are less observable by others, such as anxiety, optimism, or self-esteem (Hopwood and Bornstein, 2014; Vazire and Carlson, 2011). Within the dimensional model of PDs, this suggests that self-reports may be especially useful for assessing impairments in self-functioning and internalizing traits, such as Negative Affectivity. However, self-reports are subjective and vulnerable to biases. Individuals with PDs may have limited insight into the impact of their behavior on others (Hopwood and Bornstein, 2014; Huprich et al., 2011; Oltmanns et al., 2014; Rossi et al., 2014). Social desirability concerns may lead to underreporting of maladaptive traits (Hopwood and Bornstein, 2014; Huprich et al., 2011). Mood and other transient factors, such as fatigue, can also influence responses, with research showing that mood disorders like depression affect self-reports of both adaptive and maladaptive traits (Huprich et al., 2011). Informant reports provide a complementary perspective by capturing overt, interpersonal behaviors and socially evaluated traits, such as rudeness, talkativeness, or perceived intelligence and creativity (Hopwood and Bornstein, 2014; Vazire and Carlson, 2011). In the dimensional framework, these reports are particularly useful for assessing traits within Detachment and Antagonism domains and interpersonal functioning. Accuracy depends on the nature of the relationship between informant and target: close relationships may mirror self-report biases, whereas distant relationships may limit observation accuracy. Using multiple informants can mitigate these effects and provide a more complete assessment (Hopwood and Bornstein, 2014). Clinical interviews offer additional value by providing in-depth evaluations, allowing follow-up questions, and clarifying inconsistencies (Widiger, 2008). To avoid confirmation bias, interviews should however be semi-structured and conducted by trained clinicians (Hopwood and Bornstein, 2014). A limitation is the considerable time and resource investment required, which may affect feasibility in routine practice.

Empirical research shows that agreement between self-report, informant-report, and clinical interviews is typically low to moderate, reflecting both convergence and discrepancies (Hopwood and Bornstein, 2014). Clinical interviews tend to converge most closely with self-report measures, while both methods often yield lower rates of diagnosis compared to informant reports, which may over-report or be more sensitive to subtle traits (Oltmanns et al., 2014). For clinical purposes, both points of agreement and disagreement should be considered and integrated into treatment planning. Convergences generally indicate areas of self-awareness and can be readily addressed in feedback and therapy (Hopwood and Bornstein, 2014; Weekers et al., 2020). Discrepancies, on the other hand, may reveal limited insight and highlight areas requiring therapy focused on increasing self-awareness (Weekers et al., 2020). They may also require the clinician incorporating additional information about the patient’s relationships or life narrative to fully interpret the assessment results (Hopwood and Bornstein, 2014).

While the principles of multi-method assessment are relevant across populations, they are particularly important in older adults, who often present with complex symptom profiles and extensive life histories requiring multiple-source assessment (Penders et al., 2020; Rossi et al., 2014). Understanding how self-report, informant-report, and clinical interview measures relate to one another in this population is essential for accurate assessment and treatment planning. Most existing research, however, has focused on younger and middle-aged adults, and few studies have specifically examined these patterns within the dimensional model of PDs. Available evidence suggests that self–informant agreement may improve with age (Klonsky et al., 2002), highlighting the need for age-specific investigations. In this study, we therefore aim to examine both the convergences and discrepancies across multi-method assessments in older adults, to better understand how each method contributes uniquely and inform the clinical interpretation of PD assessments in this population.

### Present study

Prior research highlights the promise of a dimensional approach for classifying PDs across the entire adult lifespan, including older adults. In clinical settings, brief self-report instruments based on this approach could form an initial step in assessment and diagnostic formulation. However, their clinical integration currently remains limited because of limited evidence of the discriminative value of screening instruments, the absence of age- and gender sensitive normative data to accurately interpret their results and insufficient guidance on how to integrate screening instruments with other methods of assessment. This study aims to facilitate the application of self-report screening instruments of the dimensional model of PDs in clinical practice across the entire adult lifespan, and gives specific attention to older adults by:

1. Evaluating the discriminative value of the LPFS-BF 2.0 and the PID-5-BF + M in older adults, by examining their differences in a clinical versus a non-clinical sample.
2. Developing age- and gender-sensitive normative data across the adult lifespan, to establish cut-off points for mild (subclinical) and clinical pathology and evaluating these thresholds for older adults.
3. Exploring the degree of convergence and discrepancy between the LPFS-BF 2.0 and PID-5-BF + M self-reports and other assessment methods in an older clinical sample. Both measures are compared to their informant-counterparts and the LPFS-BF 2.0 is compared to the Semi-Structured Interview for Personality Functioning DSM-5 5.1 (STiP 5.1; Hutsebaut et al., 2014).

## Method

### Participants and procedure

#### Non-clinical sample

The study sample consisted of 815 participants drawn from the Belgian, Dutch-speaking general population. The respondents were recruited through multiple projects, including 398 participants from the age-neutrality study by Facon et al. (2025) and 469 additional respondents recruited by master thesis students from the Vrije Universiteit Brussel. All participants were recruited through an online survey. The online survey started with an informed consent and proceeded with a biographical questionnaire inquiring demographic variables, presented in the Supplementary material. After this, the LPFS-BF 2.0 and the PID-5-BF + M self-report questionnaires were administered. This study was approved by the Medical Ethics Committee of the University Hospital Brussels.

To ensure data quality, participants were removed from the dataset if they showed signs of inconsistent or random responding, which was measured by the Conscientious Responder Scale (Marjanovic et al., 2014) (see Instruments). This resulted in the deletion of 56 respondents. Additionally, five respondents were removed because the variable for age was missing. The final sample consisted of 754 participants comprising of 429 (56.90%) younger (<65 years old) and 325 (43.10%) older adults (65 years and older). The younger adult sample consisted of 24.70% men, 74.40% women and 0.90% nonbinary persons between 18 and 64 years old (*M* = 39.50; *SD* = 15.22). The older adults consisted of 38.50% men, 60.30% women and 1.20% non-binary persons between 65 and 97 years old (*M* = 72.47; *SD* = 6.12). Demographic results of the final sample can be found in the Supplementary material.

#### Clinical sample

The clinical sample consisted of 102 older adult patients administered at Mondriaan Topclinical Centre for Older Adults with Personality Disorders, Alexianen Zorggroep Tienen Older Adult Psychiatry, Mental Health Care Breburg PersonaCura or Public Psychiatric Care Centre Geel Division Older Adults. These are all Belgian or Dutch clinical centers, treating Dutch-speaking patients diagnosed with a variety of mental disorders such as PDs, but also mood disorders and substance-related disorders. All centers’ ethics committees approved of this study. Of the 102 patients, 47 also provided an informant version of the questionnaires and 34 participated in the STiP 5.1 interview. The total sample (*N* = 102) consisted of 52 men and 50 women between 65 and 83 years old (*M* = 70.80; *SD* = 4.65). All other demographic variables of this sample can be found in the Supplementary material.

After obtaining an informed consent, the LPFS-BF 2.0 and PID-5-BF + M self-reports were administered to all included patients, digitally or by paper-and-pencil, depending on the procedures of the center. Both methods can be combined in research design, as they have been proven to provide equivalent responses (Weigold et al., 2013). When an informant was available and both the patient and the informant gave their informed consent, informant versions of the LPFS-BF 2.0 and the PID-5-BF + M were applied. Lastly, after obtaining explicit and additional informed consent for conducting a semi-structured clinical interview, the STiP 5.1 was administered. The clinical staff assisted in selecting patients for this interview, based on whether their mental state allowed for a reliable assessment of personality. The interviews were conducted by the first author of this article or by a Master Student of the Vrije Universiteit Brussel. Both interviewers were trained in administering the STiP 5.1.

### Material

#### Level of personality functioning brief form 2.0 (LPFS-BF 2.0) (self-report and informant-report)

The LPFS-BF 2.0 is a 12-item self-report questionnaire assessing personality dysfunction based on the Alternative Model for Personality Disorders (AMPD) (Weekers et al., 2019), originally developed in Dutch. In this study, it was adapted to an informant version by converting items from first to third person (e.g., “I often do not know who I really am” to “He/she often does not know who he/she really is”). Both versions use a four-point Likert scale (1 = very/often false to 4 = very/often true), with higher scores indicating greater dysfunction. The questionnaire assesses self- and interpersonal functioning scales and yields a total impairment score by summing item responses.

Internal consistency estimates for the LPFS-BF 2.0 scales are presented in Supplementary material. Across both community and clinical samples, the self-report version demonstrated acceptable to good internal consistency for Self-Functioning, Interpersonal Functioning, and the total score. In the clinical sample, the informant-report version showed good to excellent internal consistency across all scales^1^.

#### Personality inventory for DSM-5 brief form modified plus (PID-5-BF + M) (self-report and informant report)

The Dutch PID-5-BF + M (Bach et al., 2020) is a 36-item self-report questionnaire assessing maladaptive personality traits as defined by the AMPD and ICD-11. Items are derived from the original 220-item PID-5 (Krueger et al., 2012), translated into Dutch (van der Heijden et al., 2014). As an informant version of the Dutch PID-5 exists using third-person phrasing, the PID-5-BF + M can also be administered as an informant-report (e.g., “He/she has much stronger emotional reactions than almost everyone else”). Both versions use a four-point Likert scale (0 = not at all true/often false to 3 = completely true/often true), with higher scores indicating greater maladaptive traits. The questionnaire assesses six domains (Negative Affectivity, Detachment, Disinhibition, Antagonism, Anankastia, Psychoticism), each comprising three facets measured by two items; facet and domain scores are calculated by averaging items. Although the PID-5-BF + M informant version is not yet validated, evidence supports the 220-item PID-5 informant version in younger adult clinical and community samples (Markon et al., 2013). Domain-level internal consistency coefficients are summarized in the Supplementary material. In the community sample, reliability ranged from doubtful to acceptable across domains. In the clinical sample, a similar pattern was observed; however, Antagonism showed unacceptable internal consistency and was therefore excluded from further analyses. In contrast, the informant-report version demonstrated acceptable to good reliability across all domains. Facet-level reliability estimates, based on inter-item correlations, are presented in Table 1. In the community sample, all facets demonstrated adequate to good reliability. In the clinical sample, most facets met the recommended threshold, although Deceitfulness and Perfectionism fell below acceptable levels and were excluded from further analyses. In contrast, the informant-report version showed consistently adequate to good facet-level reliability across all facets.

**Table 1:** Normative data for the LPFS-BF 2.0 scores.

*T*-score	Women		Men	
	YA	OA	YA	OA
Total-score				
≥60	29	24	28	23
≥65	32	27	31	25
Self-Functioning				
≥60	17	13	16	11
≥65	19	15	18	13
Interpersonal functioning				
≥60	13	12	14	13
≥65	15	13	15	14

Values represent the raw LPFS-BF 2.0 scores corresponding to the indicated *T*-score thresholds (≥60 and ≥65) for each demographic subgroup (YA = younger adults; OA = older adults).

#### Conscientious responder scale (CRS)

The Conscientious Responder Scale (CRS) (Marjanovic et al., 2014) was used to ensure response validity by detecting inconsistent answering in the online survey. It consists of five instructed-response items (e.g., “please choose option number five, ‘somewhat agree’”). To match the four-point Likert scale used in the LPFS-BF 2.0 and PID-5-BF + M, the response format was adjusted. Following the binomial model (Marjanovic et al., 2014), participants answering fewer than three of five items correctly were excluded. The CRS has been validated as an effective tool for distinguishing conscientious from random responders (Marjanovic et al., 2014).

#### Semi-structured interview for personality functioning DSM-5 5.1 (STiP 5.1)

The STiP-5.1 is a semi-structured clinical interview assessing personality functioning based on the DSM-5 AMPD (Hutsebaut et al., 2014). It includes 28 open questions and additional optional clarifying questions evaluating self- and interpersonal functioning across the 12 elements of the Level of Personality Functioning Scale (LPFS; American Psychiatric Association, 2013). Interviewers rate each element on a dimensional scale from 0 (no impairment) to 4 (severe impairment) based on responses. The interview takes 45–60 min and is available only in Dutch. It has demonstrated reliability and validity in clinical and community samples aged 16–61 (Hutsebaut et al., 2017), but has not yet been studied in older adults. The overall internal consistency estimates in the current study indicated acceptable to good reliability for all scales (see Supplementary material).

### Statistical analyses

First, descriptive statistics including means, standard deviations, skewness and kurtosis were computed for all included instruments in both samples.

Next, in case of normality of the data, independent sample *t*-tests were conducted for both instruments between the older adult non-clinical sample and the older adult clinical sample to evaluate their discriminative value.

Normative thresholds were derived from the non-clinical sample. Independent samples *t*-tests examined mean differences between age groups to determine whether age-specific thresholds were needed; separate thresholds were created when moderate or larger significant differences were found. Regarding gender sensitivity, because there was an unequal distribution between men and women in the sample, thresholds were calculated separately for men and women. Additionally, *t*-tests were conducted between men and women, to gather information on possible mean-level-differences. Thresholds were not developed for non-binary participants due to their small representation (1.20%).

Thresholds for the LPFS-BF 2.0 and PID-5-BF + M were defined as cut-offs at 1.5 and 1.0 SD above the latent mean, representing clinical and mild (subclinical) pathology. For interpretability, scores were converted to *T*-scores (Iverson, 2011). *T*-scores are standardized values that convert raw scores into a more interpretable format by first transforming raw scores into *z*-scores, which are then converted into *T*-scores using the formula: *T* = 50 + 10 × Z. *T*-scores have a mean of 50 and SD of 10; thus, 65 and 60 correspond to 1.5 and 1.0 SD above the mean, respectively. Next, the two cut-off points were evaluated by computing the proportion of the non-clinical sample who exceed this threshold versus those of the clinical sample and conducting *X*^2^-test to examine whether the cut-off points significantly differentiated between the two samples.

Finally, correlation and mean-level comparison of different methods of assessment were conducted to examine the degree of convergence and discrepancy between self-, informant-, and interview assessments within the clinical sample. Giving the clinical sample size, Spearman-correlations were selected to analyze associations between the self-report and the informant scales on the one hand and between the self-report and the clinical interview scales on the other hand. The following rule of thumb was applied to interpret the effect size of Spearman’s rho: ±0.10 to ±0.30 = small correlation ±0.30 to ±0.50 = medium correlation; larger than ±0.50 = large correlation (Cohen, 1998). Next, paired-sample *t*-tests compared method scores. Because the LPFS self-report and STiP-5.1 use different scales, scores were standardized (*z*-scores) prior to analysis. Effect sizes were interpreted using Cohen’s *d* (small ≥ 0.20, medium ≥ 0.50, large ≥ 0.80; Cohen, 2009), and significance was set at *p* < 0.01 due to multiple testing.

Data are publicly available via the Open Science Framework (OSF; DOI: 10.17605/OSF.IO/RMKQV; 10.17605/OSF.IO/HC9UR). Due to sensitive mental health information, only a subset of variables is shared to ensure participant anonymity.

## Results

### Descriptive results self reports (non-clinical and clinical sample)

The descriptive results (means, standard deviations, skewness and kurtosis) for the LPFS-BF 2.0 and the PID-5-BF + M self-report scales of both the non-clinical and the clinical sample are displayed in the Supplementary material.

### Mean-level differences between non-clinical and clinical sample

For the LPFS-BF 2.0, the clinical sample had significantly higher scores for all scales than the non-clinical sample, with a large effect size. For the PID-5-BF + M, all scale scores except domain Anankastia and facets Intimacy Avoidance, Manipulativeness, Grandiosity, Impulsivity, Rigidity, Orderliness and Unusual Beliefs and Experiences were found to be significantly higher in the clinical sample. Here, effect sizes ranged from small to large. All results of the independent sample *t*-tests are reported in the Supplementary material.

### Thresholds for self-reports (non-clinical sample)

#### Mean-level differences between gender–and age groups

In the non-clinical sample (consisting of younger and older adults), there were no mean-level differences between men and women on the LPFS-BF 2.0 total score. For the PID-5-BF + M, women scored significantly higher on Negative Affectivity, Emotional Liability, Anxiousness and Separation Insecurity, while men scored significantly higher on Anhedonia and Grandiosity. All these differences were of small to medium effect-size.

Younger adults scored significantly higher on the LPFS-BF 2.0 total score and on most PID-5-BF + M scales, except for domains Detachment and Anankastia and the facets Anhedonia, Intimacy Avoidance, Grandiosity, Impulsivity, Rigidity, Orderliness and Perceptual Dysregulation, where no significant differences were found. All significant differences were of small to medium effect-size. These and all other mean-level differences can be found in Tables 2, 3, along with the mean scores, 95% confidence intervals and standard errors of all subgroups.

**Table 2:** Independent sample *t*-test of the LPFS-BF 2.0 and PID-5-BF + M between men (*n* = 231) and women (*n* = 515) of the non-clinical sample.

	Men		Women			
	*M* [*CI* 95%]	*SE*	*M* [*CI* 95%]	*SE*	*t*	*d*
LPFS-BF 2.0						
*Total score*	19.71 [19.00–20.44]	0.37	20.82 [20.27–21.37]	0.28	−2.29	−0.18
PID-5-BF + M						
*Negative affectivity*	0.84 [0.77–0.90]	0.03	1.19 [1.13–1.24]	0.03	−7.96**	−0.58
Emotional lability	1.01 [0.91–1.11]	0.05	1.40 [1.33–1.48]	0.04	−6.03**	−0.46
Anxiousness	0.98 [0.88–1.09]	0.05	1.51 [1.43–1.59]	0.04	−7.96**	−0.60
Separation insecurity	0.51 [0.44–0.59]	0.04	0.65 [0.59–0.70]	0.03	−2.82*	−0.22
*Detachment*	0.63 [0.57–0.70]	0.03	0.57 [0.53–0.61]	0.02	1.63	0.13
Withdrawal	0.87 [0.78–0.96]	0.05	0.80 [0.74–0.86]	0.03	1.41	0.11
Anhedonia	0.60 [0.52–0.68]	0.04	0.46 [0.40–0.51]	0.03	3.06*	0.24
Intimacy avoidance	0.43 [0.34–0.51]	0.04	0.46 [0.40–0.53]	0.03	−0.61	−0.05
*Antagonism*	0.61 [0.55–0.66]	0.03	0.45 [0.41–0.48]	0.02	n.a.	n.a.
Manipulativeness	0.40 [0.33–0.47]	0.04	0.29 [0.25–0.33]	0.02	2.63	0.21
Deceitfulness	1.00 [0.91–1.09]	0.05	0.79 [0.73–0.85]	0.03	n.a.	n.a.
Grandiosity	0.42 [0.3–0.49]	0.04	0.26 [0.22–0.30]	0.02	3.92**	0.33
*Disinhibition*	0.75 [0.69–0.82]	0.03	0.78 [0.74–0.83]	0.02	−0.77	−0.06
Irresponsibility	0.42 [0.34–0.49]	0.04	0.33 [0.28–0.36]	0.02	2.10	0.18
Distractibility	1.00 [0.90–1.10]	0.05	1.11 [1.04–1.19]	0.04	−1.61	−0.12
Impulsivity	0.84 [0.75–0.93]	0.04	0.92 [0.85–0.98]	0.04	−1.44	−0.11
*Anankastia*	1.17 [1.10–1.24]	0.04	1.14 [1.09–1.12]	0.03	0.52	0.04
Perfectionism	1.21 [1.10–1.32]	0.05	1.16 [1.09–1.24]	0.04	n.a.	n.a.
Rigidity	1.65 [1.56–1.73]	0.04	1.69 [1.62–1.76]	0.03	−0.65	−0.05
Orderliness	0.64 [0.54–0.74]	0.05	0.58 [0.52–0.64]	0.03	1.11	0.09
*Psychoticism*	0.70 [0.64–0.76]	0.03	0.62 [0.57–0.66]	0.02	2.01	0.16
Unusual beliefs and experiences	1.09 [1.00–1.17]	0.04	0.98 [0.91–1.05]	0.03	1.85	0.14
Eccentricity	0.80 [0.70–0.90]	0.05	0.65 [0.58–0.71]	0.03	2.51	0.20
Perceptual dysregulation	0.21[0.15–0.27]	0.03	0.21 [0.17–0.25]	0.02	0.05	0.00

n.a.: insufficient reliability of the scales. *p < 0.010, **p < 0.005.

**Table 3:** Independent sample *t*-test of the LPFS-BF 2.0 and the PID-5-BF + M between younger (YA, *n* = 429) and older adults (OA, *n* = 325) in the non-clinical sample.

	Younger adults		Older adults			
	*M* [*CI* 95%]	*SE*	*M* [*CI* 95%]	*SE*	*t*	*d*
LPFS-BF 2.0						
Total score	22.27 [21.68–22.86]	0.30	18.19 [17.63–18.76]	0.29	9.79**	0.70
PID-5-BF + M						
*Negative affectivity*	1.22 [1.16–1.28]	0.03	0.89 [0.83–0.95]	0.03	7.74**	0.56
Emotional lability	1.45 [1.38–1.54]	0.04	1.05 [0.96–1.14]	0.05	6.04**	0.47
Anxiousness	1.53 [1.44–1.62]	0.04	1.12 [1.02–1.20]	0.05	6.42**	0.47
Separation insecurity	0.69 [0.63–0.75]	0.03	0.49 [0.43–0.55]	0.03	4.52**	0.33
*Detachment*	0.62 [0.57–0.66]	0.02	0.58 [0.52–0.63]	0.03	1.19	0.09
Withdrawal	0.89 [0.82–0.96]	0.03	0.75 [0.67–0.82]	0.04	2.78*	0.20
Anhedonia	0.55 [0.49–0.62]	0.03	0.45 [0.38–0.50]	0.03	2.48	0.18
Intimacy avoidance	0.41 [0.35–0.47]	0.03	0.53 [0.45–0.62]	0.04	−2.35	−0.18
*Antagonism*	0.59 [0.54–0.63]	0.02	0.37 [0.34–0.41]	0.02	n.a.	n.a.
Manipulativeness	0.46 [0.40–0.52]	0.03	0.14 [0.11–0.18]	0.02	9.34**	0.64
Deceitfulness	0.99 [0.93–1.06]	0.03	0.68 [0.60–0.75]	0.07	n.a.	n.a.
Grandiosity	0.32 [0.28–0.37]	0.03	0.30 [0.25–0.35]	0.03	0.64	0.05
*Disinhibition*	0.90 [0.85–0.95]	0.03	0.61 [0.56–0.66]	0.03	7.91**	0.57
Irresponsibility	0.46 [0.40–0.51]	0.03	0.21 [0.17–0.25]	0.02	7.18**	0.50
Distractibility	1.29 [1.22–1.38]	0.04	0.80 [0.72–0.88]	0.04	8.10**	0.60
Impulsivity	0.94 [0.87–1.01]	0.04	0.82 [0.74–0.91]	0.04	2.08	0.15
*Anankastia*	1.19 [1.13–1.24]	0.03	1.09 [1.02–1.15]	0.03	2.31	0.17
Perfectionism	1.23 [1.16–1.31]	0.04	1.09 [1.00–1.18]	0.05	n.a.	n.a.
Rigidity	1.71 [1.65–1.78]	0.03	1.62 [1.15–1.70]	0.04	1.16	0.13
Orderliness	0.62 [1.65–1.78]	0.03	0.56 [0.48–0.63]	0.04	1.32	0.10
*Psychoticism*	0.73 [0.68–0.78]	0.03	0.54 [0.49–0.59]	0.03	5.07**	0.37
Unusual beliefs and experiences	1.10 [1.03–1.18]	0.04	0.91 [0.83–0.98]	0.04	3.49**	0.26
Eccentricity	0.82 [0.75–0.90]	0.04	0.53 [0.46–0.61]	0.04	5.24**	0.38
Perceptual dysregulation	0.25 [0.25–0.30]	0.02	0.17 [0.12–22]	0.02	2.48	0.18

n.a.: insufficient reliability of the scales. *p < 0.010, **p < 0.005.

#### Identifying normative thresholds

As a result of the mean-level differences, normative data for the LPFS-BF 2.0 was developed for younger and older adults, separately. For the PID-5-BF + M, mean-level differences were found between men and women and between younger and older adults, resulting in the development of separate thresholds for gender–and age groups. The complete overview of the normative thresholds for these scales are presented in Tables 1, 4, 5.

**Table 4:** Normative data for the PID-5-BF + M domains.

	Women		Men		Women		Men	
	YA	OA	YA	OA	YA	OA	YA	OA
*T*-score	Negative affectivity				Detachment			
≥60	1.93	1.52	1.42	1.25	1.06	0.97	1.15	1.04
≥65	2.24	1.82	1.68	1.49	1.30	1.20	1.39	1.26
*T*-score	Disinhibition				Antagonism			
≥60	1.39	1.10	1.47	0.98	1.77	0.62	1.24	0.78
≥65	1.66	1.35	1.75	1.19	2.07	0.78	1.48	0.95
*T*-score	Anankastia				Psychoticism			
≥60	1.77	1.64	0.96	1.73	1.19	1.00	1.35	0.95
≥65	2.07	1.95	1.46	2.02	1.46	1.25	1.61	1.15

Values represent the raw PID-5-BF+M domain scores corresponding to the indicated *T*-score thresholds (≥60 and ≥65) for each demographic subgroup (YA = younger adults; OA = older adults).

**Table 5:** Normative data for the PID-5-BF + M facets.

	Women		Men		Women		Men	
	YA	OA	YA	OA	YA	OA	YA	OA
*T*-score	Emotional liability				Anxiety			
≥60	2.38	1.92	1.79	1.74	2.53	2.08	1.91	1.58
≥65	2.80	2.36	2.18	2.12	2.98	2.51	2.33	1.94
*T*-score	Separation insecurity				Withdrawal			
≥60	1.37	1.05	1.15	0.97	1.57	1.23	1.48	1.61
≥65	1.70	1.32	1.46	1.24	1.93	1.54	1.81	1.97
*T*-score	Anhedonia				Intimacy avoidance			
≥60	1.11	0.89	1.41	0.97	1.00	1.35	1.09	1.04
≥65	1.43	1.15	1.76	1.20	1.32	1.75	1.43	1.37
*T*-score	Manipulative behavior				Deceitfulness			
≥60	0.91	0.46	1.30	0.49	1.54	1.19	1.89	1.45
≥65	1.19	0.63	1.63	0.66	1.87	1.51	2.24	1.78
*T*-score	Grandiosity				Irresponsibility			
≥60	0.71	0.65	1.01	0.88	0.92	0.57	1.26	0.64
≥65	0.93	0.87	1.31	1.12	1.18	0.76	1.60	0.84
*T*-score	Impulsivity				Distractibility			
≥60	1.67	1.68	1.69	1.29	2.15	1.56	1.98	1.50
≥65	2.05	2.09	2.07	1.59	2.60	1.96	2.37	1.87
*T*-score	Perfectionism				Rigidity			
≥60	2.05	1.81	1.94	2.04	2.40	2.42	2.29	2.27
≥65	2.47	2.23	2.34	2.46	2.75	2.84	2.62	2.61
*T*-score	Orderliness				Unusual beliefs and experiences			
≥60	1.28	1.14	1.34	1.36	1.80	1.58	1.94	1.53
≥65	1.62	1.47	1.72	1.73	2.19	1.95	2.30	1.84
*T*-score	Eccentricity				Perceptual dysregulation			
≥60	1.50	1.13	1.79	1.29	0.68	0.59	0.83	0.49
≥65	1.89	1.48	2.21	1.63	0.91	0.81	1.10	0.67

Values represent the raw PID-5-BF+M facet scores corresponding to the indicated T-score thresholds (≥60 and ≥65) for each demographic subgroup (YA = younger adults; OA = older adults).

#### Evaluating cut-off scores

The *X*^2^-test showed that the *T* ≥ 65 cut-off significantly differentiated clinical and non-clinical participants in both men and women for the LPFS-BF 2.0 total score and for the PID-5-BF + M domains of Negative Affectivity, Detachment, Disinhibition and Pyschoticism (*X*^2^ ranged from 3.94 to 32.23, with *p* < 0.01 to < 0.001). The cut-off of *T* ≥ 65 for Anankastia did not significantly differentiate the sample, for neither genders.

The cut-off of *T* ≥ 60 significantly differentiated between clinical and non-clinical participants for the LPFS-BF 2.0 total score and the PID-5-BF + M domain of Detachment for both men and women (*X*^2^ ranged from 11.76 to 46.20, with *p* < 0.001). Only in men, a cut-off of T ≥ 60 for Disinhibition and Psychoticism also significantly differentiated the two samples (*X*^2^ = 21.75; *p* < 0.001 and *X*^2^ = 6.86; *p* < 0.01, respectively), while the T ≥ 60 cut-off for Negative Affectivity was only significant for women (*X*^2^ = 31.01; *p* < 0.001). The cut-off of T ≥ 60 for Anankastia did not significantly differentiate the sample, for neither genders (*X*^2^ = 0.01, 0.15; *p* > 0.01). Antagonism was not included in the analyses due to poor reliability in the clinical sample. Detailed results of the *X*^2^-tests can be found in the Supplementary material.

### Multi-method evaluation (clinical population)

#### Descriptive results informant report and clinical interview

The descriptive results (means, standard deviations, skewness and kurtosis) for the LPFS-BF 2.0 and the PID-5-BF + M informant-report scales and the STiP 5.1 scales of the clinical sample are displayed in the Supplementary material.

#### Comparisons multi-method

Significant, positive Spearman correlations were found between the LPFS-BF 2.0 self- and informant reports for the Total Scores (medium effect size) and the Self-Functioning scores (medium effect size). For the PID-5-BF + M significant, positive correlations emerged between self- and informant ratings for the domains of Negative Affectivity, Disinhibition and Anankastia and the facets of Emotional Liability, Separation Insecurity, Withdrawal, Irresponsibility and Impulsivity (medium to large effect sizes). The LPFS-BF 2.0 self-report correlated significantly with all STiP 5.1 scales, all positive and of medium effect size. These and all other correlations can be found in Table 6.

**Table 6:** Spearman correlations between the different evaluation methods.

Self-Report	(*n* = 47)	(*n* = 34)
	Informant-report	Clinical interview
Personality functioning		
*Total score*	0.41*	0.45*
Self-functioning	0.55**	0.48*
Interpersonal-functioning	0.28	0.45*
Maladaptive personality traits		
*Negative affectivity*	0.59**	n.a.
Emotional lability	0.72**	n.a.
Anxiousness	0.36	n.a.
Separation insecurity	0.56*	n.a.
*Detachment*	0.34	n.a.
Withdrawal	0.49**	n.a.
Anhedonia	0.26	n.a.
Intimacy avoidance	0.35	n.a.
*Antagonism*	n.a.	n.a.
Manipulativeness	−0.06	n.a.
Deceitfulness	n.a.	n.a.
Grandiosity	0.00	n.a.
*Disinhibition*	0.40*	n.a.
Irresponsibility	0.56**	n.a.
Distractibility	0.25	n.a.
Impulsivity	0.38*	n.a.
*Anankastia*	0.38*	n.a.
Perfectionism	n.a.	n.a.
Rigidity	0.37	n.a.
Orderliness	0.28	n.a.
*Psychoticism*	0.18	n.a.
Unusual beliefs and experiences	0.16	n.a.
Eccentricity	0.25	n.a.
Perceptual dysregulation	−0.05	n.a.

n.a. = not applicable: insufficient reliability or no available interview; *p < 0.010, **p < 0.005.

The paired-sample *t*-tests between the self- and informant report version of the LPFS-BF 2.0 showed that there were no significant differences on any of the three personality functioning scores. Similarly, no significant differences were found between the three scales of the LPFS-BF 2.0 self-report and the STiP 5.1. Regarding the self- and informant report version of the PID-5-BF + M, a marginally significant result of a small effect size was found for the Unusual Beliefs and Experiences facet. Tables 7–9 display an overview of these results.

**Table 7:** Paired sample *t*-tests between the self- and informant report version of the LPFS-BF 2.0 (*n* = 47).

LPFS-BF 2.0	Self-report *M* (*SD*)	Informant Report *M* (*SD*)	*t*	Cohen’s *d*
Total-score	26.96 (8.12)	26.57 (9.00)	0.29	0.04
Self-Functioning	14.06 (4.57)	13.23 (4.94)	1.25	0.18
Interpersonal Functioning	12.89 (4.34)	13.34 (4.98)	−0.57	−0.08

All found differences were non-significant.

**Table 8:** Paired sample *t*-tests between the LPFS-BF 2.0 Self-Report and the STiP 5.1 (*n* = 34) (Sumscores).

Personality functioning	Self-report *M* (*SD*)		Clinical Interview *M* (*SD*)		*t*	Cohen’s *d*
	Raw Score	*z*-score	Raw Score	*z-*score		
Total-score	24.63	−0.09	13.91	0.00	−0.52	−0.09
Self-functioning	12.53	−0.07	8.00	0.00	−0.40	−0.07
Interpersonal functioning	12.10	−0.09	5.91	0.00	−0.49	−0.08

*z*-scores were used in the paired-sample *t*-tests. All found differences were non-significant.

**Table 9:** Paired sample *t*-tests between the self- and informant report version of the PID-5-BF + M (*n* = 47).

Maladaptive personality traits	Self-report *M* (*SD*)	Informant report *M* (*SD*)	*t*	Cohen’s *d*
*Negative affectivity*	1.58 (0.73)	1.56 (0.84)	0.18	0.03
Emotional lability	1.74 (0.98)	1.63 (1.10)	1.00	0.15
Anxiousness	1.83 (0.95)	1.79 (1.03)	0.27	0.04
Separation insecurity	1.15 (0.85)	1.28 (0.94)	−0.98	−0.14
*Detachment*	1.04 (0.68)	1.01 (0.72)	0.25	0.04
Withdrawal	1.21 (0.84)	1.13 (0.94)	0.66	0.10
Anhedonia	1.16 (0.92)	1.07 (0.96)	0.51	0.07
Intimacy avoidance	0.74 (0.94)	0.82 (0.97)	−0.49	−0.07
*Antagonism*	0.49 (0.41)	0.58 (0.64)	n.a.	n.a.
Manipulativeness	0.22 (0.49)	0.31 (0.62)	−0.82	−0.12
Deceitfulness	0.72 (0.49)	0.74 (0.93)	n.a.	n.a.
Grandiosity	0.53 (0.61)	0.72 (0.79)	−1.28	−0.19
*Disinhibition*	1.13 (0.70)	1.04 (0.68)	0.72	0.11
Irresponsibility	0.53 (0.82)	0.76 (0.86)	−1.85	−0.27
Distractibility	1.49 (0.82)	1.23 (0.98)	1.37	0.20
Impulsivity	1.35 (0.93)	1.15 (0.94)	1.28	0.19
*Anankastia*	1.21 (0.77)	1.16 (0.87)	0.34	0.05
Perfectionism	1.07 (0.83)	0.95 (1.03)	n.a.	n.a.
Rigidity	1.73 (0.89)	1.46 (0.97)	1.83	0.27
Orderliness	0.81 (0.98)	1.08 (0.90)	−1.66	−0.25
*Psychoticism*	0.90 (0.64)	0.63 (0.55)	2.39	0.35
Unusual beliefs and experiences	1.26 (0.90)	0.83 (0.78)	2.69*	0.39
Eccentricity	0.95 (0.89)	0.79 (0.79)	1.01	0.15
Perceptual dysregulation	0.49 (0.72)	0.27 (0.53)	1.62	0.24

*p = 0.010.

## Discussion

The present study focused on enhancing the implementation of a dimensional model for personality disorders (PDs) in clinical practice across the entire adult lifespan, with a special focus on older adults. This model defines PDs through two core criteria: impairments in personality functioning and the presence of maladaptive personality traits. First, the potential of the LPFS-BF 2.0 and PID-5-BF + M as screening instruments for personality functioning and traits, respectively, was evaluated by examining their ability to differentiate between a clinical and a non-clinical sample. Next, age- and gender-specific thresholds, were established and evaluated for both instruments. Finally, recognizing the importance of a multi-method approach in PD assessment, especially in older adults, the study also compared these self-report measures with informant reports and clinical interviews.

To be clinically relevant in the screening procedure, measures should be able to differentiate between patient samples and other non-clinical samples. This has been demonstrated for the LPFS-BF 2.0 before, as the study by Juras et al. (2025) showing that its scores are predictive for being treatment-seeking. The current study further supports the discriminative value of the measure in older adults, as all LPFS-BF 2.0 scales (total, self and interpersonal) were significantly lower in the non-clinical sample than in the clinical sample. Regarding the PID-5-BF + M, a more specific picture emerged. Not all of the domains and facets yielded higher scores in the clinical sample. More specifically, mainly the domain Anankastia and its facets (Perfectionism, Rigidity, Orderliness) showed not to vary significantly across the clinical versus the non-clinical sample. This is contrary to the earlier study by Pires et al. (2023), where all domains and facets showed to be higher in the clinical PD sample. One explanation for this may be that the current study employed a sample of clinical patients with a variation of mental disorders, with or without PD, while the Pires et al. (2023) sample consisted solely of patients diagnosed with a PD. This may have caused differences on certain traits to be bigger when compared to a PD sample, assuming trait levels are higher in PD specific samples. Additionally, the Pires et al. (2023) study included both younger and older adults, suggesting that age may also influence the discriminative value of the PID-5-BF + M. All other maladaptive domains and facets included in the analyses did yield higher scores in the clinical sample, indicating that the PID-5-BF + M does capture clinically significant scores in older adults for most scales. In light of the broader literature on the incremental validity between personality functioning and pathological traits (e.g., Facon et al., 2026; Thimm, 2025; Valls et al., 2023), the pattern observed here, where gender differences are present for traits but not for overall functioning, may support the idea that these constructs do seem to capture (at least some) unique aspects of personality pathology, with gender playing a different role in each of them.

As a next step, thresholds for dysfunctioning were developed to identify exact thresholds for the distinction between subclinical and clinically elevated scores across the adult lifespan, based on the non-clinical sample. Because previous findings have showed that age and gender can influence PD aspects (e.g., Facon et al., 2025; Le Corff et al., 2023), this study prioritized sensitivity to these factors. Consistent with earlier research (Facon et al., 2025), younger adults in the current study scored significantly higher on the personality pathology measures, with higher scores on the LPFS-BF 2.0 total score and on most of the PID-5-B + M scales. This motivated the decision to develop thresholds separately for the younger versus the older adult age group. These mean-level age differences are reflected in Tables 1, 4, 5, as the normative thresholds for younger adults are lower than those for older adults. Regarding gender differences, women scored significantly higher on traits within the Negative Affectivity domain, while men yielded significantly higher scores on traits mainly within the Antagonism domain, as well as on the facet of Anhedonia. These findings are consistent with previous research on gender differences in the PID-5 (Le Corff et al., 2023). Even though no significant gender differences were found for the LPFS-BF 2.0, it was still decided to develop gender-specific thresholds for both instruments, given that the current sample was not stratified for age. These findings were also reflected in the normative thresholds, as the cut-offs for maladaptive traits vary according to gender, with for example higher cut-off scores in men for Antagonism and in women for Negative Affectivity. As there were no significant gender differences in the LPFS-BF 2.0, these normative thresholds are more similar for men and women.

The normative thresholds were set at 1.0 (T ≥ 60) and 1.5 (T ≥ 65) standard deviations above the latent mean, which is generally considered to correspond to mild and moderate personality dysfunctioning and/or traits, forming the cut-off between subclinical and clinical levels of personality pathology. Evaluation of these thresholds showed that for the LPFS-BF 2.0 total score, and for the PID-5-BF + M domains of Detachment, both the mild and the moderate thresholds are able to differentiate between clinical and non-clinical older adults, for both men and women. This suggests that even “mild” evaluations in these domains may be an indication for clinically relevant personality pathology in older adults. For Disinhibition and Psychoticism, the cut-offs of *T* ≥ 65 differentiated significantly in both men and women, but the *T* ≥ 60 cut-offs only did so for men. This may mean that personality pathology in women may be less frequently expressed through these two domains, making them less clinically significant than for men. Conversely, Negative Affectivity traits seem to be more clinically relevant for women than for men, as the *T* ≥ 60 cut-off only differentiated significantly for women, while the *T* ≥ 65 did for both genders. For Anankastia, neither one of the cut-offs showed significant differentiation, in men nor in women. This may be due to Anankastia-related traits, such as perfectionism, being common in non-clinical populations compared to other maladaptive traits. Especially in older adults, both the current study and prior research has shown Anankastia to be the most prevalent trait among community-dwelling samples (Facon et al., 2025). This higher presence in non-clinical samples reduces the distinction with clinical samples, diminishing its discriminative value. Summarized, when implementing the dimensional model for PDs in clinical practice, clinicians should pay attention to demographic factors. These findings suggest that diagnostic accuracy may improve with differentiated thresholds, as uniform cut-off scores may lead to loss of important information and under- or over-diagnosing. Additionally, it is essential to consider the clinical relevance of the PID-5-BF + M traits, as they did all not hold the same discriminative value.

Because it is well known that accurate PD diagnosis in older adults requires multi-method assessment (Penders et al., 2020; Rossi et al., 2014), it is essential to understand how different assessment methods relate to one another in this age group. As this topic has thus far only been evaluated in general adult samples (Oltmanns et al., 2014; Vazire and Carlson, 2011), the current study conducted an exploratory examination of these relationships in older adults. For personality functioning, associations were around 0.50 between self-report and informant-report instruments of the Total Scale and the Self-Functioning Scale, but no significant association was found for the Interpersonal-Scale Between the self-report and the STiP 5.1 clinical interview, similar size association were found, but in this case for all three scales. This suggests that self-reports and clinical interviews have the strongest correspondence, consistent with earlier findings (Oltmanns et al., 2014). However, since there were no significant differences found between any of the methods for personality functioning, correspondence between the three methods seems higher than in earlier studies with younger adults (aged between 55 and 64 years old) (Oltmanns et al., 2014). This aligns with prior results suggesting that the self-other correspondence becomes stronger with age (Klonsky et al., 2002). For clinical practice, this may suggest that in the case of issues with self-report data in older patients (e.g., due to limited energy), informant-ratings can be used to form an assessment for personality functioning.

Regarding maladaptive personality traits, significant correlations were observed between self- and information reports for Negative Affectivity, Disinhibition and Anankastia. The domains that did not show significant correlations were Detachment and Psychoticism. Antagonism was excluded from the analyses due to poor reliability, however its reliable facets (Manipulativeness and Grandiosity) also did not show significant correlations. The Detachment domain and Antagonism facets are characterized by social and interpersonal aspects such as keeping people at a distance or deserving special treatment from others, which may be experienced and rated differently by the individual than by their close ones (Vazire and Carlson, 2011). Nonetheless, just like with personality functioning, no significant differences were found on these domains between the two methods, again suggesting a higher self-other correspondence with age. The only significant difference found between self- and informant report was for the Unusual Beliefs and Experiences facet, with self-reports scoring slightly higher than informant ratings. It may be so that psychotic-like experiences in the form of beliefs and experiences are inherently more internal, less visible or recognizable for informants and less openly talked about by the individuals themselves. Another interesting observation concerns the Perfectionism facet, which showed to be reliably measured by the informant version of the PID-5-BF + M, but not by its self-report version. At first glance, this may be surprising, because perfectionism is often viewed as an internalized process. However, one of the two items capturing perfectionism in the PID-5-BF + M involves the perspective of others, as it states that the individual insists on doing everything perfectly, even if it drives other people crazy (item 6). It may be possible that the individual has a less accurate view on this item than others rating the person, leading to the difference in reliability.

In summary, multi-method agreement of the dimensional aspects of personality in older adults seems to vary according to the aspect measured. Even though the results point to higher convergence at an older age, addressing the discrepancies remains important, as some may be more apparent to others than to the individual. Clinicians can use these divergences to identify blind spots and enhance self-awareness and thus inform treatment planning (Weekers et al., 2020).

### Limitations and considerations

The current study consisted of a number of limitations. First, it is important to note that the developed thresholds are based on self-report data, which could be influenced by biases and lack of self-insight. Therefore, self-report data should be complemented by additional assessment methods, of which age and gender sensitive thresholds should also be made available. Next, future studies should corroborate the current thresholds in larger, more stratified samples, to conclude official norms. In the future, it may be valuable to divide participants into more narrow age groups, allowing for more specific age-sensitivity. Additionally, as the data was derived from a Belgian sample, the thresholds are only applicable to the Belgian population, especially as previous research suggests established different normative thresholds for the LPFS-BF 2.0 in different populational samples (Juras et al., 2025; Weekers et al., 2022).

Next, the clinical sample in this study included patients with a range of mental disorders, and not all patients had a PD diagnosis. Moreover, a dimensional diagnostic gold standard for personality disorders in older adults is currently lacking. As a result, it was not possible to classify participants as having or not having a PD, which limited the analyses. In future research, the availability of such a gold standard would allow for more comprehensive evaluation of the instruments, including the examination of sensitivity and specificity, as well as the incremental predictive value of the assessment methods relative to the gold standard. Additionally, future studies could repeat the analyses with a PD-specific sample, which may alter the findings. For instance, it would be valuable to examine whether individuals with PDs score higher on the LPFS-BF 2.0 and PID-5-BF + M compared to those with other clinical conditions, and whether the associations between different assessment methods shift when applied within a PD-specific group. Furthermore, because a Dutch-language clinical interview for maladaptive traits currently does not exist at this moment, the PID-5-BF + M self- and interpersonal reports could not be compared to a clinical interview in the current study. Future research into the dimensional model and clinical application would benefit from language variations of this instrument. Another limitation in the clinical sample concerns the insufficient reliability of the Antagonism domain, excluding it from the analyses conducted in this sample. This was also reported by previous studies in older adults (Debast et al., 2018; Facon et al., 2025). Because this finding seems consistent, further research seems necessary to improve the domain’s reliability across the lifespan. Lastly, the clinical sample size was relatively small and only included older adults, which limits statistical power and the generalizability of the findings. Replication with larger clinical samples is needed to validate the current exploratory results.

## Data Availability

The raw data supporting the conclusions of this article will be made available by the authors, without undue reservation.

## Footnotes

1 These and all other Omega coefficients were evaluated using the same threshold values as those used for Cronbach’s Alpha: >0.70 = acceptable; >0.80 = good; >0.90 = excellent (Nunnally and Bernstein, 1994).
