# Supplementary material for: Facilitating clinical application of a dimensional model for personality disorders across the entire adult lifespan: thresholds for dysfunction and a multi-method approach
Source: Front Psychol. 2026 Apr 24;17:1734213. doi: 10.3389/fpsyg.2026.1734213 (PMC13152793; doi:10.3389/fpsyg.2026.1734213)
Supplement: Supplementary file 1 [file Table_1.DOCX]

**Supplemental Material**

**Table 1**

Demographics of the Non-clinical Sample (*N* = 754).

| Demographic Variables | Younger Adults (YA)  (*n* = 429) | Older Adults (OA)  (*n* = 325) |
| --- | --- | --- |
| Age |  |  |
| Range | 18-64 | 65-97 |
| Mean (SD) | 39.50 (15.22) | 72.47 (6.12) |
| Gender |  |  |
| Male | 24.70% | 38.50% |
| Female | 74.4% | 60.30% |
| Nonbinary | 0.90% | 1.20% |
| Nationality |  |  |
| Belgian | 98.60% | 98.80% |
| Other | 1.40% | 1.20% |
| Civil status |  |  |
| Married | 35.00% | 60.90% |
| Single | 30.90% | 5.80% |
| Legally divorced | 7.50% | 12**.**60% |
| Living together | 9.80% | 2.20% |
| Widowed | 1.60% | 15.10% |
| Blended family after divorce or death ddddof a spouse | 1.20% | .30% |
| Other | 1.60% | 3.10% |
| Living Situation (YA) |  |  |
| Partner | 54.80% | N/A |
| Children | 30.50% | N/A |
| Parents | 25.90% | N/A |
| Siblings | 14.20% | N/A |
| Alone | 12.60% | N/A |
| Other | 4.20% | N/A |
| Living Situation (OA) |  |  |
| Independent living | N/A | 94.20% |
| Service flat | N/A | 1.20% |
| Retirement home | N/A | 1.20% |
| Other | N/A | .30% |
| Missing | N/A | 3.10% |
| Highest education level |  |  |
| Primary | .90% | 1.50% |
| Lower secondary | 4.00% | 15.10% |
| Higher secondary | 28.90% | 23.10% |
| Higher vocational | 3.30% | 4.90% |
| Higher education | 29.40% | 33.70% |
| University | 33.60% | 19.70% |
| Consulting a mental health professional |  |  |
| Currently | 14.70% | 3.10% |
| During last three years (BE) | 30.80% | 8.90% |

*Note.* N/A = not applicable. The YA and OA subsamples used two distinct demographical surveys regarding their living situations. Moreover, the variable options for living situation were not mutually exclusive.

**Table 2**

Demographics of the Clinical Sample (*N* = 102).

| Demographic Variables |  |
| --- | --- |
| Age |  |
| Range | 65-83 |
| Mean (SD) | 70.80 (4.65) |
| Gender |  |
| Male | 51.00% |
| Female | 49.00% |
| Nonbinary | 0.00% |
| Nationality |  |
| Belgian | 46.10% |
| Dutch | 53.90% |
| Civil status |  |
| Married | 44.10% |
| Single | 14.70% |
| Legally divorced | 10.80% |
| Living together | 0.00% |
| Widowed | 11.80% |
| Other | 0.00% |
| Missing | 18.6% |
| Highest education level (BE) |  |
| Primary | 15.70% |
| secondary | 36.30% |
| Higher vocational | 12.70% |
| University | 2.00% |
| Missing | 33.30% |
| Primary Psychiatric Diagnosis |  |
| Personality Disorder | 16.70% |
| Anxiety Related Disorder | 2.90% |
| Depression | 13.7% |
| Bipolar Disorder | 1.00% |
| Attention Deficit Hyperactivity Disorder | 5.90% |
| Autism Spectrum Disorder | 6.90% |
| Psychotrauma and Stress-Related Disorders | 6.90% |
| Dissociative Disorder | 1.00% |
| Obessive Compulsive Disorder | 1.00% |
| Substance-Related Disorder | 21.60% |
| Other | 9.80% |
| Missing | 12.70% |
| Medication Use |  |
| No Medication | 11.80% |
| Antidepressants and/or Mood-Stabilizers | 15.70% |
| Benzodiazepines/Hypnotics | 20.60% |
| Antipsychotics | 7.80% |
| Stimulants | 0.00% |
| Other | 8.80% |
| Missing | 50.00% |

*Note.* The variable options for medication use were not mutually exclusive.

**Table 3**

Descriptive Statistics of Self-Reports in the Non-Clinical and the Clinical Sample and Results of the Independent *t*-test between the Older Adult Non-Clinical Sample and the Clinical Sample .

|  | Non-clinical Sample (*N* = 754) | | | | | | Clinical Sample (N = 102) | | | | Independent *t*-test | |
| --- | --- | --- | --- | --- | --- | --- | --- | --- | --- | --- | --- | --- |
|  | Younger Adults (*n* = 429) | | | Older Adults (*n* = 325) | | | Older Adults | | | |  |  |
|  | *M (SD)* | *S* | *K* | *M (SD)* | *S* | *K* | | *M (SD)* | *S* | *K* | *t* | Cohen’s *d* |
| LPFS-BF 2.0 |  |  |  |  |  |  |  | |  |  |  |  |
| *Total score* | 22.27 (6.24) | .40 | -.28 | 18.19 (5.18) | .96 | .44 | 24.63 (7.88) | | .24 | -.98 | -7.74** | -1.08 |
| Self | 12.25 (4.02) | .24 | -.80 | 8.88 (3.12) | 1.24 | 1.04 | 12.53 (4.73) | | .23 | -1.01 | -7.31** | -1.02 |
| Interpersonal | 10.02 (3.04) | .80 | .62 | 9.31 (2.74) | .81 | .11 | 12.10 (4.18) | | .47 | -.51 | -6.32** | -.89 |
| PID-5-BF+M |  |  |  |  |  |  |  | |  |  |  |  |
| *Negative Affectivity* | 1.22 (.63) | .16 | -.49 | .89 (.56) | .51 | -.37 | 1.32 (0.77) | | .31 | -.84 | -5.23** | -.70 |
| Emotional Lability | 1.45 (.86) | -.03 | -.91 | 1.05 (.83) | .43 | -.80 | 1.46 (1.04) | | .04 | -1.15 | -3.57** | -.46 |
| Anxiousness | 1.53 (.92) | -.04 | -1.02 | 1.11 (.83) | .32 | -.86 | 1.50 (1.00) | | .08 | -1.22 | -3.55** | -.45 |
| Seperation Insecurity | .69 (.65) | .92 | .78 | .49 (.55) | 1.00 | .36 | 1.00 (.87) | | .52 | -.75 | -5.46** | -.79 |
| *Detachment* | .62 (.49) | .92 | .77 | .68 (.47) | .92 | .58 | .96 (0.63) | | .51 | -1.81 | -5.56** | -.75 |
| Withdrawal | .99 (.70) | .45 | -.57 | .75 (.68) | .59 | -.52 | 1.07 (0.83) | | .18 | -1.13 | -3.49** | -.44 |
| Anhedonia | .55 (.66) | 1.09 | .54 | .45 (.53) | 1.18 | 1.19 | 1.06 (0.86) | | .43 | -.71 | -6.67** | -.98 |
| Intimacy Avoidance | .41 (.65) | 1.71 | 2.45 | .53 (.77) | 1.50 | 1.55 | .76 (0.95) | | 1.05 | -.08 | -2.14 | -.27 |
| *Antagonism* | .59 (.44) | .73 | .14 | .37 (.33) | .95 | 1.00 | .44 (0.40) | | 1.01 | .70 | n.a. | n.a. |
| Manipulativeness | .46 (.59) | 1.12 | .33 | .14 (.34) | 2.67 | 7.18 | .23 (0.46) | | 2.34 | 6.03 | -1.67 | -.23 |
| Deceitfulness | .99 (.68) | .27 | -.60 | .68 (.65) | .74 | -.07 | .65 (0.67) | | .75 | -.25 | n.a. | n.a. |
| Grandiosity | .32 (.49) | 1.44 | 1.28 | .30 (.47) | 1.75 | 2.96 | .46 (0.61) | | 1.56 | 2.84 | -2.32 | -.30 |
| *Disinhibition* | .90 (.54) | .39 | -.21 | .61 (.47) | .67 | -.00 | .95 (0.68) | | .65 | .21 | -4.66** | -.65 |
| Irresponsibility | .46 (.57) | 1.26 | 1.05 | .21 (.40) | 1.85 | 2.63 | .52 (0.73) | | 1.47 | 1.62 | -4.09** | -.63 |
| Distractibility | 1.29 (.87) | .17 | -.83 | .80 (.78) | .71 | -.37 | 1.31 (1.00) | | .27 | -.93 | -4.68** | -.61 |
| Impulsivity | .94 (.76) | .44 | -.59 | .82 (.75) | .64 | -.40 | 1.05 (0.93) | | .63 | -.60 | -2.17 | -.28 |
| *Anankastia* | 1.19 (.59) | .27 | -.25 | 1.09 (.61) | .23 | -.63 | 1.04 (0.68) | | .71 | .24 | .62 | .07 |
| Perfectionism | 1.23 (.83) | .25 | -.77 | 1.09 (.85) | .31 | -.91 | .95 (0.78) | | .64 | -.10 | n.a. | n.a. |
| Rigidity | 1.71 (.69) | -.37 | .08 | 1.62 (.77) | -.42 | -.37 | 1.58 (0.94) | | .06 | -1.02 | .38 | .05 |
| Orderliness | .62 (.70) | 1.09 | .67 | .56 (.70) | 1.08 | .29 | .60 (0.83) | | 1.39 | 1.09 | -.46 | -.06 |
| *Psychoticism* | .73 (.54) | .70 | .03 | .54 (.46) | 1.22 | 1.83 | .72 (0.60) | | .87 | -.00 | -2.76* | -.36 |
| Unusual Beliefs and Experiences | 1.10 (.78) | .31 | -.64 | .91 (.69) | .47 | -.40 | 1.05 (0.84) | | .54 | -.65 | -1.43 | -.18 |
| Eccenctricity | .82 (.80) | .74 | -.26 | .53 (.69) | 1.27 | .95 | .79 (0.87) | | .64 | -.88 | -2.71* | -.35 |
| Perceptual Dysregulation | .25 (.49) | 2.07 | 3.91 | .17 (.42) | 2.96 | 9.31 | 0.32 (0.60) | | 1.97 | 3.29 | -2.34 | -.32 |

*Note. S* = Skewness; *K* = Kurtosis.

n.a.: insufficient reliability of the scales.

* *p* < .01., ** *p* ≤ .001.

**Table 4**

Proportion of Older Adults in the Non-Clinical (N-C) versus Clinical (C) Sample Exceeding the Cut-off of T ≥ 65.

|  | Men | |  | Women | |  |
| --- | --- | --- | --- | --- | --- | --- |
| Scale | N-C Sample | C Sample | Chi Square | N-C | C Sample | Chi Square |
| **LPFS-BF 2.0** |  |  |  |  |  |  |
| Total Score | 10.4% | 38.5% | 19.07** | 11.2% | 46% | 32.23** |
| **PID-5-BF+M** |  |  |  |  |  |  |
| Negative Affectivity | 10.4% | 28% | 8.48* | 11.2% | 40% | 23.30** |
| Detachment | 8.8% | 28% | 10.75* | 8.7% | 36% | 24.38** |
| Disinhibition | 8% | 40% | 25.75** | 6.1% | 18% | 7.20* |
| Antagonism | 10.4% | n.a. | n.a. | 9.2% | n.a. | n.a. |
| Anankastia | 5.6% | 6% | .01 | 10.7% | 6% | 1.01 |
| Psychoticism | 12% | 24% | 3.94* | 8.7% | 22% | 7.01* |

*Note*. n.a.: insufficient reliability of the scale.

* *p* < .01.

** *p* ≤ .001

**Table 5**

Proportion of Older Adults in the Non-Clinical (N-C) versus Clinical (C) Sample Exceeding the Cut-off of T ≥ 60.

|  | Men | |  | Women | |  |
| --- | --- | --- | --- | --- | --- | --- |
| Scale | N-C Sample | C Sample | Chi square | N-C Sample | C Sample | Chi square |
| **LPFS-BF 2.0** |  |  |  |  |  |  |
| Total Score | 19.2% | 50% | 17.19** | 14.3% | 60% | 46.20** |
| **PID-5-BF+M** |  |  |  |  |  |  |
| Negative Affectivity | 17.6% | 32% | 4.36 | 16.3% | 54% | 31.01** |
| Detachment | 14.4% | 40% | 13.77** | 20.4% | 44% | 11.76** |
| Disinhibition | 21.6% | 58% | 21.75** | 20.4% | 24% | .31 |
| Antagonism | 16.8% | n.a. | n.a. | 17.3% | n.a. | n.a. |
| Anankastia | 20.8% | 20% | .01 | 21.4% | 24% | .15 |
| Psychoticism | 17.6% | 36% | 6.86* | 16.8% | 30% | 4.40 |

*Note*. n.a.: insufficient reliability of the scale.

**p* < .01.

** *p* ≤ .001

**Table 6**

Descriptives of the Informant-Reports and Clinical Interview in the Clinical Sample (*N* = 102).

|  | Informant-Report (*n* = 47) | | | Clinical Interview (*n* = 34) | | |
| --- | --- | --- | --- | --- | --- | --- |
|  | *M (SD)* | *Skewness* | *Kurtosis* | *M (SD)* | *Skewness* | *Kurtosis* |
| Personality Functioning | |  |  |  |  |  |
| *Total score* | 26.90 (9.18) | -.08 | -.68 | 14.19 (7.63) | .71 | -.25 |
| Self | 13.355 (4.95) | .24 | -1.05 | 8.16 (4.12) | .44 | -.82 |
| Interpersonal | 13.54 (5.12) | .00 | -.33 | 6.03 (4.28) | .61 | -.43 |
| Maladaptive Personality Traits | |  |  |  |  |  |
| *Negative Affectivity* | 1.56 (.84) | .15 | -.97 | n.a. | n.a. | n.a. |
| Emotional Lability | 1.63 (1.10) | -.01 | -1.03 | n.a. | n.a. | n.a. |
| Anxiety | 1.79 (1.03) | -.13 | -1.44 | n.a. | n.a. | n.a. |
| Seperation Insecurity | 1.28 (.94) | 1.20 | -.98 | n.a. | n.a. | n.a. |
| *Detachment* | 1.56 (.84) | .48 | -.78 | n.a. | n.a. | n.a. |
| Withdrawal | 1.13 (.94) | .32 | -1.06 | n.a. | n.a. | n.a. |
| Anhedonia | 1.07 (.96) | .46 | -1.06 | n.a. | n.a. | n.a. |
| Intimacy Avoidance | .82 (.97) | .86 | -.65 | n.a. | n.a. | n.a. |
| *Antagonism* | .58 (.64) | 1.53 | 2.40 | n.a. | n.a. | n.a. |
| Manipulativeness | .31 (.62) | 2.63 | 7.69 | n.a. | n.a. | n.a. |
| Deceitfulness | .74 (.93) | 1.10 | .28 | n.a. | n.a. | n.a. |
| Grandiosity | .72 (.79) | 1.23 | 1.20 | n.a. | n.a. | n.a. |
| *Disinhibition* | 1.04 (.68) | .09 | -.71 | n.a. | n.a. | n.a. |
| Irresponsibility | .75 (.86) | .95 | -.23 | n.a. | n.a. | n.a. |
| Distractibility | 1.13 (.98) | .06 | -1.32 | n.a. | n.a. | n.a. |
| Impulsivity | 1.15 (.94) | .32 | -1.07 | n.a. | n.a. | n.a. |
| *Anankastia* | 1.16 (.87) | .44 | -.70 | n.a. | n.a. | n.a. |
| Perfectionism | .95 (1.03) | .58 | -1.23 | n.a. | n.a. | n.a. |
| Rigidity | 1.46 (.97) | -.10 | -1.10 | n.a. | n.a. | n.a. |
| Orderliness | 1.07 (.90) | .71 | -.19 | n.a. | n.a. | n.a. |
| *Psychoticism* | .63 (.55) | .74 | -.51 | n.a. | n.a. | n.a. |
| Unusual Beliefs and Experiences | .83 (.78) | .68 | -.54 | n.a. | n.a. | n.a. |
| Eccenctricity | .79 (.79) | .85 | -.38 | n.a. | n.a. | n.a. |
| Perceptual Dysregulation | .27 (.53) | 2.43 | 6.49 | n.a. | n.a. | n.a. |

*Note.* n.a. = not applicable: no available interview.
